# The carnivorous digestive system and bamboo diet of giant pandas may shape their low gut bacterial diversity

**DOI:** 10.1093/conphys/coz104

**Published:** 2020-03-13

**Authors:** Wei Guo, Yinfeng Chen, Chengdong Wang, Ruihong Ning, Bo Zeng, Jingsi Tang, Caiwu Li, Mingwang Zhang, Yan Li, Qingyong Ni, Xueqin Ni, Hemin Zhang, Desheng li, Jiangchao Zhao, Ying Li

**Affiliations:** 1 Department of Laboratory Medicine, School of Laboratory Medicine/Sichuan Provincial Engineering Laboratory for Prevention and Control Technology of Veterinary Drug Residue in Animal-origin Food, Chengdu Medical College, 783 Xindu Road, Xindu, Chengdu, Sichuan, 610500, China; 2 Department of Animal Science, Farm Animal Genetic Resources Exploration and Innovation Key Laboratory of Sichuan Province, Sichuan Agricultural University, 211 Huimin Road, Wenjiang, Chengdu, Sichuan, 611130, China; 3 Department of Feeding, China Conservation and Research Center for the Giant Panda, 155 Xianfeng Road, Ya’an, Sichuan, 611830, China; 4 Department of animal medicine, College of Veterinary Medicine, Sichuan Agricultural University, 211 Huimin Road, Wenjiang, Chengdu, Sichuan, 611130, China; 5 Department of Animal Science, Division of Agriculture, University of Arkansas, 2404 North University Avenue Little Rock, Fayetteville, Arkansas, 72207, USA

**Keywords:** bamboo diet, carnivorous digestive system, dietary switch, diversity, gut microbiotas

## Abstract

- The gut microbiota diversity of eight panda cubs was assessed during a dietary switch.
- Gut microbiota diversity of panda cubs significantly decreased after bamboo consumption.
- Carnivorous species living on a plant-based diet possess low microbial diversity.
- Mice were fed a bamboo diet but did not display low gut microbiota diversity.

Giant pandas have an exclusive diet of bamboo; however, their gut microbiotas are more similar to carnivores than herbivores in terms of bacterial composition and their functional potential. This is inconsistent with observations that typical herbivores possess highly diverse gut microbiotas. It is unclear why the gut bacterial diversity of giant pandas is so low. Herein, the dynamic variations in the gut microbiota of eight giant panda cubs were measured using 16S rRNA gene paired-end sequencing during a dietary switch. Similar data from red panda (an herbivorous carnivore) and carnivorous species were compared with that of giant pandas. In addition, mice were fed a high-bamboo diet (80% bamboo and 20% rat feed) to determine whether a bamboo diet could lower the gut bacterial diversity in a non-carnivorous digestive tract. The diversity of giant panda gut microbiotas decreased significantly after switching from milk and complementary food to bamboo diet. Carnivorous species living on a plant-based diet, including giant and red pandas, possess a lower microbial diversity than other carnivore species. Mouse gut microbiota diversity significantly increased after adding high-fibre bamboo to their diet. Findings suggest that a very restricted diet (bamboo) within a carnivorous digestive system might be critical for shaping a low gut bacterial diversity in giant pandas.

## Introduction

Despite living on a bamboo-dominated diet, the giant panda lacks genes for bamboo digestion (Li *et al.*, 2010). Therefore, the giant panda gut microbiota plays a putative role in obtaining nutrients from bamboo (Zhu *et al.*, 2011, Wei *et al.*, 2015); however, recent studies have revealed that the gut microbiota of giant pandas may be unsuitable for a bamboo diet (Li *et al.*, 2015, Xue *et al.*, 2015, Guo *et al.*, 2018, Zhang *et al.*, 2018). The bacterial diversity of the herbivore gut is significantly greater than that of omnivores and carnivores (carnivore < omnivore < herbivore) (Ley *et al.*, 2008). A high-fibre diet can significantly increase the bacterial diversity of the human and animal gut (Sonnenburg *et al.*, 2016). Giant pandas are unique members of the order Carnivora, as they have diverged to consume a predominantly plant-based diet. The diet of giant pandas consists of almost 99% bamboo (Schaller *et al.*, 1985), and they have a lower gut bacterial diversity than other animals (Xue *et al.*, 2015, McKenney *et al.*, 2018). However, it is unclear why the giant panda’s gut bacterial community displays low species diversity. Considering that giant panda possess a typical and simple carnivore-like short gastrointestinal tract (Dierenfeld *et al.*, 1982, Schaller *et al.*, 1985), we propose the hypothesis that ‘a bamboo diet with a carnivorous digestive system shapes the low gut bacterial diversity in giant pandas’.

During growth and development (0~1.5 year old), giant pandas in captivity change from a diet of breast and formula milk and supplementary food (e.g. steamed corn bread, carrot and fruits) to bamboo. Herein, to investigate why a low-diversity bacterial community exists in giant pandas, a 16S rRNA gene deep-sequencing study of their gut microbiota was performed when switching them from a milk-based to bamboo-based diet. This could determine whether all bamboo specialists that have evolved from a carnivorous diet display a lower gut bacterial diversity than other carnivores. To test this hypothesis, the gut microbiotas of giant pandas, red pandas and other species of selected carnivores were compared. In addition, an experiment was performed on mice to test if a bamboo diet in a non-carnivorous digestive system leads to a gut bacterial community with low diversity.

## Materials and methods

### Sample collection

This study was approved by the Institutional Animal Care and Use Committee of Sichuan Agricultural University under permit number DKY-B20130302. Eight captive giant panda cubs, including four males and four females born within the same week from eight different mother pandas, were selected to survey the giant panda gut microbiome dynamics in early life. All giant panda cubs lived together with their mother from birth to 8 mos; they were subsequently housed in a separate house with large yard. A series of faecal samples were collected from the eight giant panda cubs (samples were taken once a month from 4 to 17 mos) and 31 adult giant pandas (> 5 years) from the China Conservation and Research Center for the Giant Panda (Ya’an, Sichuan Province, China). The giant panda enclosure was broad and complex and panda cubs often entered bushes for a week, so there may have been some impact on regular sample collection times. Diet and antibiotic usage were recorded. All samples were stored at −80°C until use.

### DNA extraction, PCR amplification and 16S rRNA gene sequencing

A frozen aliquot (200 mg) of each sample was processed using an MO BIO Power Faecal TM DNA Isolation Kit (MO BIO Laboratories, Carlsbad, CA, USA) according to the manufacturer’s protocol. The DNA concentration was measured using a NanoDrop Spectrophotometer (Thermo Scientific), and the overall DNA quality was assessed by agarose gel electrophoresis. Only samples that met the following criteria were used for sequencing: (i) DNA concentration > 10 ng/ul and (ii) DNA total quantity > 100 ng. Polymerase chain reaction (PCR) amplification and paired-end sequencing of the 16S V4 region (250 bp length) were performed by the Beijing Genomics Institute (BGI, Shenzhen, China) using an Illumina MiSeq platform. Briefly, the V4 hypervariable region of the bacterial 16S rRNA gene was amplified from extracted DNA using standard barcoded primers (515F: GTGCCAGCMGCCGCGGTAA, 806R: GGACTACHVGGGTWTCTAAT) (Caporaso *et al.*, 2011). Genomic DNA was normalized to 30 ng per PCR reaction. V4 Dual-index Fusion PCR Primer Cocktail and PCR Master Mix (NEB Phusion High-Fidelity PCR Master Mix) were then added, and PCR was performed using the following thermocycling parameters: 95°C for 3 min, 30 cycles of 95°C for 45 s, 56°C for 30 s and 72°C for 90 s, and a final extension at 72°C for 10 min. The PCR products were purified using AmpureXP beads (AGENCOURT).

**Figure 1:**
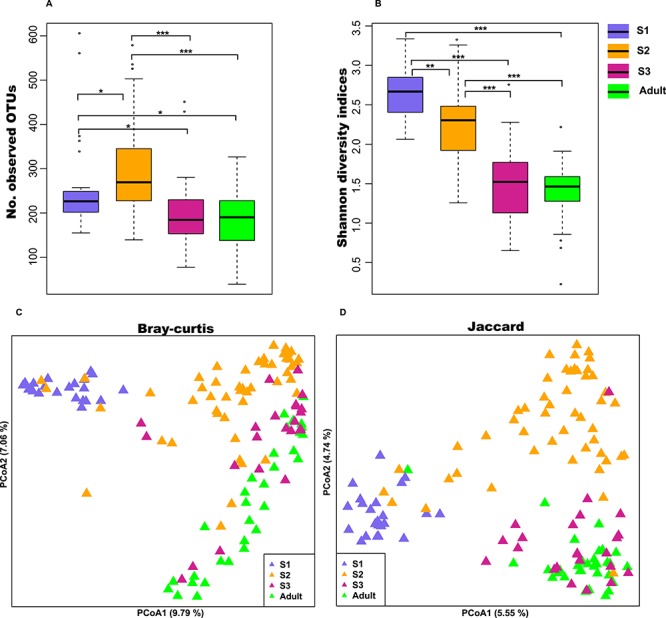
Alpha and beta diversity of the gut microbiota of the different growth stages of giant pandas. (A) Number of observed OTUs. (B) Shannon diversity indices. Principal coordinate of (C) Bray-Curtis and (D) Jaccard distance. ^*^P < 0.05, ^**^P < 0.01 and ^***^P < 0.001 by Mann-Whitney U test.

**Figure 2:**
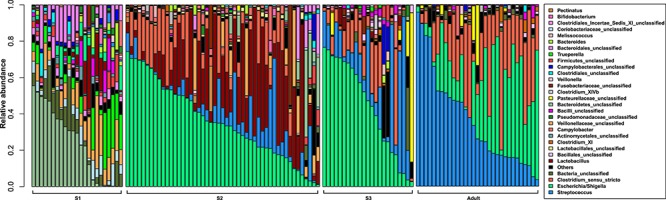
Relative abundance of the top 30 OTUs at the genus level in the faecal microbiota of giant pandas at different growth stages.

### 16S sequence processing and analysis

To compare the gut bacterial diversity between giant pandas and carnivores, published paired-end data for the 16S V4 region from carnivores were used. High-throughput sequencing data for the V4 region of the 16S rDNA gene of red panda (Williams *et al.*, 2018), black bear (Song *et al.*, 2017), cat (Bermingham *et al.*, 2018), black-backed jackal (Menke *et al.*, 2014), cheetah (Menke *et al.*, 2014), dhole (Wu *et al.*, 2016) and leopard cat (An *et al.*, 2017) were downloaded for comparative analysis (see Table S1 for dataset information). Datasets were obtained using the same sequencing technology as the present study (paired-end sequencing using a 16S V4 region, Miseq platform). Evidence from previous studies suggests that the giant panda harbours a bacterial community with lower diversity than other herbivores (Xue *et al.*, 2015, McKenney *et al.*, 2018).

The Mothur v1.39 software package was used to process and analyse 16S rDNA paired-end raw reads while referring to standard operating procedures (SOP) of the Miseq platform (Kozich *et al.*, 2013). Quality filtering was performed according to the following criteria: sequences were discarded with (i) ambiguous bases (quality score of Q ≥ 20); (ii) homo-polymers longer than 8 bp; and (iii) length greater than 300 bp or less than 250 bp. Clean reads were then aligned using the full-length SILVA reference database (release 128, http://www.arb-silva.de/). Chimeric sequences were excluded using the VSEARCH algorithm (Rognes *et al.*, 2016). Furthermore, sequences assigned to chloroplasts were removed. Operational taxonomic units (OTUs) were determined with a threshold of 97% identity, and singleton OTUs were discarded to reduce the sequencing error. Data were normalized by the lowest number of reads (19,000) obtained in a given sample to calculate the alpha and beta diversity metrics. Alpha diversity was estimated using observed OTUs and Shannon index. Jaccard and Bray-Curtis distances were also used to explore the structure of gut communities of giant pandas at different growth stages. In addition, we focused on the variation of the abundance of dominant bacterial community (the top 30) by significance tests between two groups (S1 vs S2; S1 vs S3; S2 vs S3).

### High-bamboo diet experiment in mice

To determine if a bamboo diet is a predominant factor reducing the alpha diversity of the giant panda gut microbiota, an experiment was performed on mice (Fig. 6A). All mice were single-housed in stainless steel cages. Bamboo leaves and stems were ground into powder using an electric grinder, and a bamboo diet was prepared by mixing the bamboo powder with rat feed at the ratios 50:50% and 80:20% with boiling water. A total of 20 Kunming (KM) mice were used for high-bamboo diet experiment. Ten KM male mice (9 weeks) were fed a 50% bamboo diet (50% bamboo and 50% rat feed) for 7 days. The mice were then fed a high-bamboo diet (80% bamboo and 20% rat feed) from days 8 to 28. From days 29 to 56, mice were fed rat feed only. As a control group, 10-KM male mice (9 weeks) were fed rat feed during the entire experiment (see Table S2 for rat feed composition). Faecal samples were collected from all mice on day 0 (prior to the experiment), 7, 14, 28, 35, 42 and 56. The procedures for DNA extraction, PCR amplification, 16S rRNA gene sequencing and data analysis were performed as described earlier.

### Statistical analysis

Figures were drawn using R packages. Tests of significance were based on the Mann-Whitney U test and were used to determine significant differences in bacterial diversity and composition in all results using GraphPad Prism 7 software (GraphPad Software, Inc., USA).

**Figure 3:**
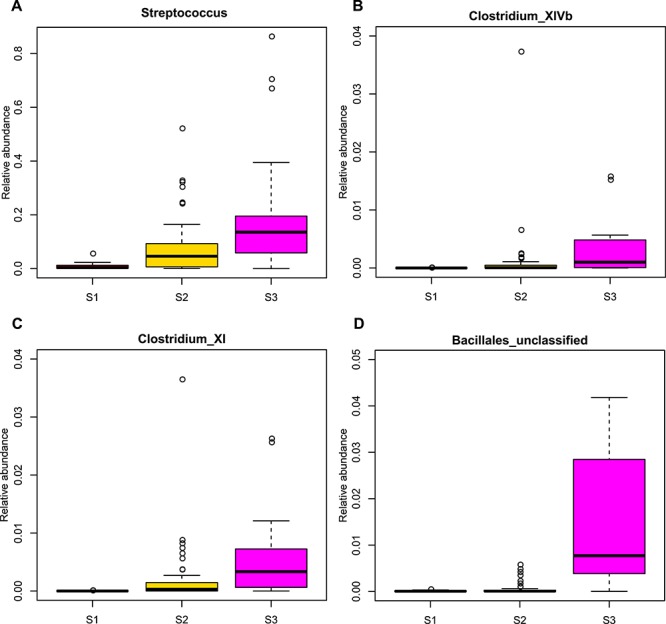
Distributions of relative abundances are shown as box plots for gut bacteria that significantly increased in group S3. (A) *Streptococcus*, (B) *Clostridium_XlVb*, (C) *Clostridium*_XI and (D) *Bacillales*_unclassified.

**Figure 4:**
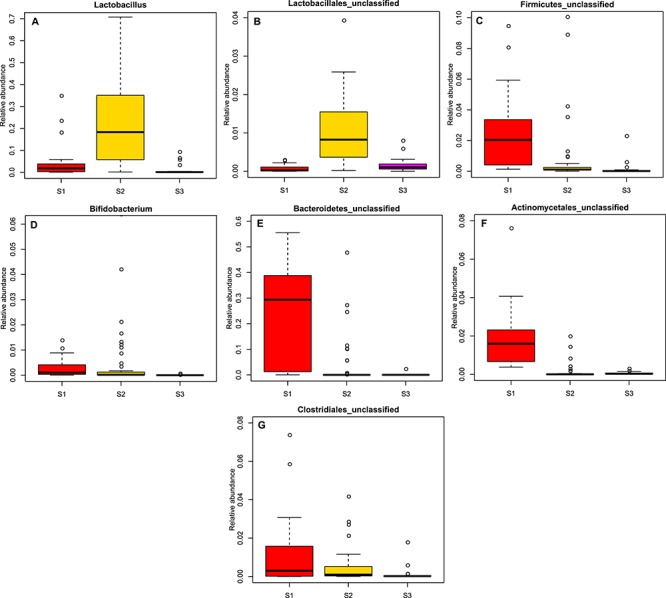
Distributions of relative abundances are shown as box plots for gut bacteria that significantly reduced in group S3. (A) *Lactobacillus*, (B) *Lactobacillales*_unclassified, (C) *Firmicutes*_unclassified, (D) *Bifidobacterium*, (E) *Bacteroidetes*_unclassified, (F) *Actinomycetales*_unclassified and (G) *Clostridiales*_unclassified.

## Results

### 16S rRNA gene sequencing data

Sequencing data were obtained for eight giant panda cubs (4–17 mos) and 31 adult pandas (>5 years). Giant panda cubs were categorized as S1, S2 and S3 based on their diet (Table S3). S1 (4–7 mos) were characterized as milk-fed (dominant) with supplementary food (steamed corn bread, carrots and fruit), S2 (8–13 mos) as milk-fed with supplementary food (dominant) and bamboo leaves, and S3 (14–17 mos) as bamboo stem or leaf-fed only. After filtering ambiguous bases and low-quality sequences, 4 936 942 valid reads were obtained, which varied from 21 751 to 61 600 per sample. Sequencing of four samples failed due to low DNA concentration; sequencing libraries were not constructed from these samples (see Table S4 for sex, age and sequencing information).

### Gut microbiota structure and diversity of giant pandas at different stages of growth

After basic data processing using Mothur (v1.39), 4 936 942 high-quality reads were assigned to 6443 OTUs with a threshold of 97% similarity. A Venn diagram (Fig. S1) shows that S2 had more unique OTUs (*n* = 1953), followed by S1 (*n* = 476) and S3 (*n* = 326). Unexpectedly, adult giant pandas had the fewest unique OTUs (*n* = 276) and only 359 OTUs were present in all growth stages (including 16.76% in S1, 7.79% in S2, 15.43% in S3 and 17.32% in adults).

The alpha diversities, as indicated by observed OTUs (Fig. 1A) and Shannon index (Fig. 1B), were significantly lower in adult and S3 stages than in S1 and S2 stages (*P* < 0.05). The dynamic variation of observed OTUs (Fig. S2) and Shannon index (Fig. S3) in the eight giant panda cubs also showed a similar trend (although the curve was very volatile, it eventually dropped to the lowest point after an exclusive bamboo diet was introduced). In addition, principal coordinate analysis (PCoA) based on Bray-Curtis (Fig. 1C) and Jaccard (Fig. 1D) distances demonstrated that the overall gut microbiota structures of S1, S2 and S3 were distinct from each other, with S3 clustering closer to that of the adult giant panda. Here, the result of Principal coordinate analysis in Fig. 1C and 1D explain only a small fraction of the total variance.

Giant panda cubs were found to have completely different gut microbiotas at different stages of growth. Gut microbial communities displayed greater variation between individuals in group S1 compared with groups S2 and S3, which were dominated by *Escherichia/Shigella*, *Streptococcus* and *Escherichia/Shigella*, respectively (Fig. 2 and Fig. S4). *Bacteroidetes*_unclassified, *Coriobacteriaceae*_unclassified, *Veillonellaceae*_unclassified and *Trueperella* were the predominant genera in group S1. Results suggest that the structure of the panda gut microbiota depends on their dominant diet (Fig. 2). Due to their exclusive bamboo diet, group S3 displayed a similar gut microbiota to the adult group (Fig. 2). In terms of the composition of gut bacteria, the relative abundance of *Streptococcus*, *Clostridium*_XlVb, *Clostridium*_XI and *Bacillales*_unclassified increased significantly in group S3 (Fig. 3, Table S5), and *Lactobacillus*, *Lactobacillales*_unclassified, *Firmicutes*_unclassified, *Bifidobacterium*, *Bacteroidetes*_unclassified, *Actinomycetales*_unclassified and *Clostridiales*_unclassified decreased significantly in group S3 (Fig. 4, Table S5).

To determine which factors significantly affect the development of gut microbial communities in giant panda cubs, permutational multivariate analysis of variance (PERMANOVA) was used on diet, individual, genetics, sex, season and age data (see Table 1 for the differences in these factors). Among these, diet, genetics and season significantly correlated with the gut microbial communities of giant panda cubs (Table 1, *P* < 0.05), with diet being the most influential (*F* = 12.9142).

### Alpha diversity of the gut microbiota in bamboo specialists and carnivores

**Figure 5:**
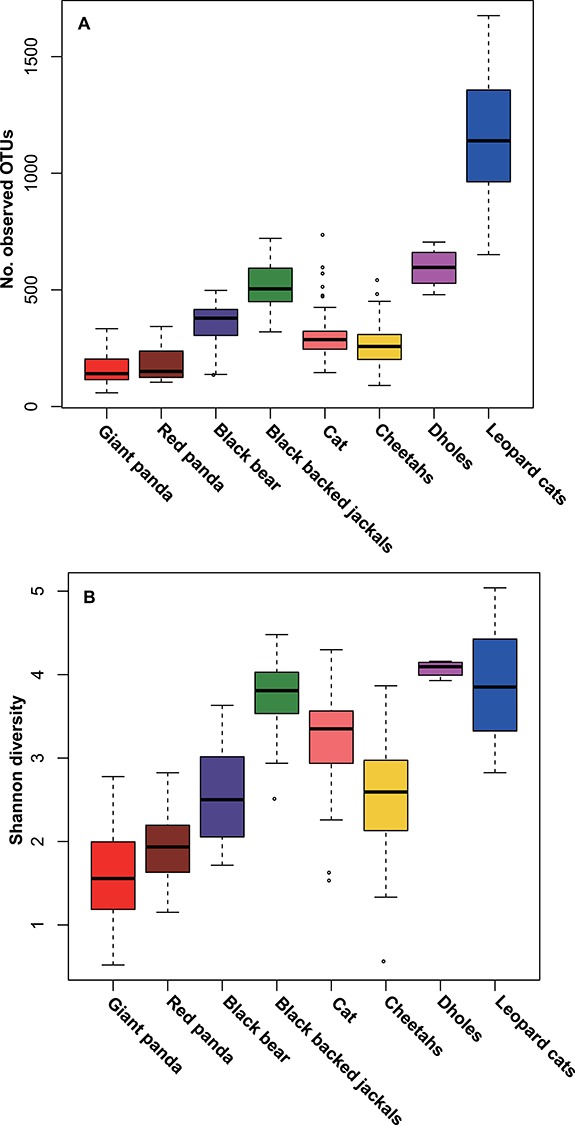
Comparisons of (A) the number of observed OTUs and (B) Shannon diversity indices among giant pandas, red pandas and carnivores.

To verify whether bamboo specialists that have evolved from a carnivorous diet harbour a lower gut bacterial diversity than carnivores, we compared the gut microbiotas of giant pandas which mainly live on bamboo (including 31 individual adult specimens and 23 specimens from group S3) with carnivores. Significantly, lower numbers of OTUs (Fig. 5A) and Shannon index (Fig. 5B) values were observed in bamboo specialists (Mann Whitney test, *P* < 0.05) compared with specific carnivorous and omnivorous representatives. Unsurprisingly, there was no significant difference between the bamboo specialists—the giant panda (*Ailuropoda melanoleuca*) and red panda (*Ailurus fulgens*) (Mann Whitney test, *P* > 0.05).

### Animal testing

**Figure 6:**
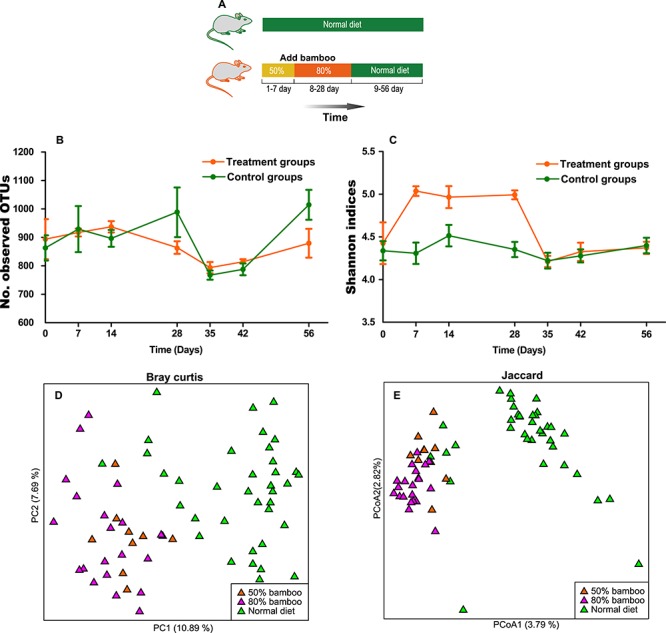
Alpha and beta diversity of the gut microbiota of mice. (A) Schematic diagram of the mice experiment. (B) Dynamic curve of the Observed OTUs in gut microbiota of the experimental and control groups. (C) The Shannon indices dynamic curve of gut microbiota in the experimental group and control group. PCoA based on (D) Bray Curtis and (E) Jaccard distance.

An experiment was performed on mice to investigate if alpha diversity decreases when bamboo is added to their diet (Fig. 6A). Altogether, 136 stool samples were obtained in the mice experiment. From these samples, 16S rRNA gene sequencing generated 9 429 433 high-quality sequences (Table S6). DNA total quality of four samples did not meet the criteria (>100 ng) for library preparation. High-quality data were not obtained for these four samples (Table S6). Contrary to giant panda results, mice alpha diversities, as indicated by observed OTUs (Fig. 6B) and Shannon index (Fig. 6C), did not show a downtrend after introducing bamboo. In fact, the Shannon index significantly increased after bamboo was introduced (Mann Whitney test*, P* < 0.05), which subsequently returned to the original level when rat feed was reintroduced (Fig. 6C). However, after the introduction of bamboo, the gut communities of bamboo-fed mice were distinct from mice fed exclusively with rat feed (Mann Whitney test, *P* < 0.05) (Fig. 6D and E). Not surprisingly, experimental mice slightly lost weight when they were fed a bamboo diet, and restored the weight upon return to a normal diet (Fig. S5). The low nutrition of a bamboo diet is likely to be the main reason for this observation.

**Table 1:** PERMANOVA pseudo-F and p-values associated with specific factor

Factors	Variables (Groups)	Bray–Curtis	
		pseudo-F	p-value
Diet	3 (milk(dominant) and supplementary foods/milk , supplementary foods (dominant) and bamboo leaves/ definitely bamboo stems or leaves)	12.9142	0.001
Family	8	1.5036	0.014
Age	4 (S1/S2/S3/adult)	1.7695	0.071
Gender	2 (male/female)	1.3844	0.156
Season	4 (spring/summer/autumn/winter)	5.8619	0.001

^*^number of permutations: 999.

## Discussion

Almost all tested mammals, including humans (Stewart *et al.*, 2018), mice (Pantoja-Feliciano *et al.*, 2013), cows (Jami *et al.*, 2013), sika deers (Li *et al.*, 2018), pigs (Chen *et al.*, 2017) and dogs (Guard *et al.*, 2017), have shown a change in alpha diversity after weaning. Unexpectedly, we found that the alpha diversity of giant pandas decreased when consuming a bamboo dominant diet (from milk and complementary food to bamboo) (Fig. 1A and B). A similar study declared that the number of observable OTUs of gut microbiotas in giant pandas increased with age (Zhang *et al.*, 2018). In their study, Zhang *et al.* divided giant panda cubs into four groups: S1 (<2 mos); S2 (between 3 and 12 mos and no bamboo in faeces); S3 (>6 mos and bamboo stems or leaves in faeces); and S4 (>6 mos and bamboo shoots in faeces). We found that their partial sample collection age of S2, S3 and S4 overlapped; for example, the age range of group S2 was 3–12 mos, and the age range of groups S3/S4 was 6–24 mos. However, in our study, cub faeces were divided into different groups according to diet and age (completely different ages) (Table S3). This may be the reason why the conclusions of these two studies were different. A further examination of the study by Zhang *et al.* revealed that group S2 (3–12 mos and no bamboo in faeces) had the higher Shannon diversity indices than group S3 (>6 mos and bamboo stems or leaves in faeces) and group S4 (>6 mos and bamboo shoots in faeces) (Fig. S5D, (Zhang *et al.*, 2018). In addition, giant pandas had the largest amount of observable OTUs before consuming bamboo (between 9 and 11 mos) [Fig. S5A, (Zhang *et al.*, 2018)], which is consistent with our results (Fig. S2).

The composition of gut microbiotas in giant pandas was influenced by diet. For example, the relative abundance of *Lactobacillus* and *Bifidobacterium* decreased significantly after weaning in group S3. It has been reported that *Lactobacillus* and *Bifidobacterium* positively correlate with breast milk consumption (Chen *et al.*, 2017, Li *et al.*, 2018, Stewart *et al.*, 2018). In addition, when giant panda cubs (group S3) were only fed bamboo, the relative abundance of *Clostridium*_XlVb and *Clostridium*_XI significantly increased. We speculated that these two bacteria may be play an important role in digesting cellulose. This conclusion is similar to a previous study which suggested that cellulose and hemicellulose-digesting genes are found in species within the *Clostridium* genus (Zhu *et al.*, 2011).

Similarly to the gut microbiota of giant pandas, red panda (*A. fulgens*, Carnivora: Musteloidea) adults also demonstrate a lower alpha diversity of gut bacteria (bamboo, leaf eater diet) compared with cubs during weaning (bamboo introduced) and post-weaning (early stage of bamboo diet) (Williams *et al.*, 2018). Despite the highly fibrous nature of bamboo, giant and the red pandas, unexpectedly, have a lower gut bacterial diversity than that of other carnivores (Fig. 5A and B). This result is contrary to previous evidence that: (i) the gut bacterial diversity of herbivores is significantly greater than that of omnivores and carnivores (Ley *et al.*, 2008) and (ii) dietary fibre increases the diversity of microbial communities in humans and other animals (Makki *et al.*, 2018) (Sonnenburg *et al.*, 2016, Liu *et al.*, 2018). Several studies have reported that bamboo has antimicrobial activity (Chuyen *et al.*, 1982, Nishina *et al.*, 1991). Thus, it is possible that the observed decrease in the alpha diversity of giant panda gut microbiomes after feeding on bamboo might be due to antimicrobial activity.

To test the possibility of bamboo antimicrobial activity on gut microbiomes, a bamboo feed experiment was performed on a mouse model. Contrary to the giant and red panda observations, we found that the Shannon index of mouse gut microbiotas significantly increased after adding bamboo to their diet (Fig. 6C). This result is consistent with the common observation that a high-fibre diet increases the diversity of microbial communities in mammals (Sonnenburg *et al.*, 2016, Liu *et al.*, 2018, Makki *et al.*, 2018). Despite the differences in mouse and panda gastrointestinal tract structure, metabolism and lifestyle, we speculate that animals that possess a typical omnivorous digestive system may not lose gut bacterial diversity when changing their diet to a high-bamboo diet (80% bamboo and 20% rat feed). In addition, the bamboo lemur (*Hapalemur griseus*), a primate bamboo specialist with an omnivorous digestive tract, showed significantly greater gut microbiota diversity compared with the two bamboo specialists (the giant and red pandas) evolved from carnivores (McKenney *et al.*, 2018). It seems that a bamboo diet with a non-carnivorous digestive system does not support a low-diversity gut bacterial community. Over millions of years, giant pandas have successfully evolved from carnivores to bamboo-eating herbivores, but they still possess a straight, short and simple gastrointestinal tract (Davis, 1964). When giant pandas change their diet to low-energy bamboo, their carnivorous digestive tract is not adapted to a specialized bamboo diet in the following ways: (i) to degrade low-nutrition fibre, herbivore guts have evolved to shape a rumen or an enlarged cecum; however, the giant panda still lacks these physiological structures and (ii) long transit times are needed to ferment fibre in the herbivore digestive tract; however, subject to a short and simple gastrointestinal tract, the transit time of bamboo in the giant panda gastrointestinal tract is very short (Dierenfeld *et al.*, 1982, Schaller *et al.*, 1985), and this has been considered to affect the diversity of their gut microbiota (McKenney *et al.*, 2018). A high gut diversity would not be expected in a species with such a short retention time of bamboo and a very restricted diet. The diversity of gut microbiota in animals is associated with dietary diet diversity and type (Katherine et al. 2013, Mckenzie *et al.*, 2017). Therefore, we speculate that a carnivorous digestive system fed almost exclusively on bamboo may be the reason that giant pandas have the lowest gut microbiota diversity compared with other mammals (Fig. 5A and B).

A recent study has reported that the relative abundances of genes involved in cellulose and hemicellulose digestion were significantly lower in the faeces of giant pandas than other herbivores (Guo *et al.*, 2018, Zhang *et al.*, 2018). There has been increasing evidence that the gut microbiotas of giant pandas may not adapt to their bamboo diet (Li *et al.*, 2015, Xue *et al.*, 2015, Guo *et al.*, 2018, Zhang *et al.*, 2018). In our study, the findings regarding the diversity of gut microbiotas in giant pandas suggest that a carnivorous digestive system fed with a bamboo-dominated diet is not a typical feature of evolution. A high-fibre bamboo diet with a carnivorous digestive system develops a very low-diverse bacterial community. Chemical signatures from giant panda fossils indicate how bears that feed exclusively on bamboo could have developed 5000 years ago, rather than 2 million years ago (Han *et al.*, 2019). This could explain the evidence showing that the gut microbiota of giant pandas is not adapted to their diet (Xue *et al.*, 2015, Guo *et al.*, 2018). However, giant pandas have successfully evolved in ecological, morphological and genetic terms to adapt to their diet (Wei *et al.*, 2015). In the future, additional studies of giant panda metatranscriptomes, metaproteomes and metabolomes could reveal the role of the gut microbiota of giant pandas in adapting to a bamboo diet.

## Conclusion

We found that the diversity of the giant panda gut microbiota significantly decreases when cubs begin an exclusive bamboo diet and also that bamboo specialists (giant and red pandas) harbour a lower gut bacterial diversity than other carnivores. However, a high-bamboo diet in a non-carnivorous digestive tract (mice and bamboo lemur) does not lead to the development of low gut bacterial diversity. This suggests that a very specialized diet with a carnivorous digestive system establishes a low-diversity bacterial community in giant and red pandas.

## Ethics approval and consent to participate

This study was approved by the Institutional Animal Care and Use Committee of the Sichuan Agricultural University under permit number DKY-B20130302.

## Consent for publication

All authors read and approved the submission of this article.

## Availability of data and material

The dataset used in this study was deposited into the National Centre for Biotechnology Information’s Sequence Read Archive under accession bioproject number: PRJNA524253.

## Funding

This work was supported by the National Natural Science Foundation of China (31900307) to W.G and School-level fund of chengdu medical college (18Z171) to W.G.

## Authors’ contributions

W.G. and Y.L. designed the study. W.G., R.N, J.T, C.W., H.Z., C.L. and D.L. collected the samples. W.G., R.N., Y.C and B.Z. performed the laboratory work. M.Z., Y.L., Q.N. and X.N. contributed the experimental design. W.G and J.Z. analysed the data. W.G. and Y.L. wrote the article.

## Supplementary Material

figure_s1_coz104

figure_s2_coz104

figure_s3_coz104

figure_s4_coz104

figure_s5_coz104

table_s1_coz104

table_s2_coz104

table_s3_coz104

table_s4_coz104

table_s5_coz104

table_s6_coz104

supplemental_file_legends_coz104
