# Supplementary figures and images for: The carnivorous digestive system and bamboo diet of giant pandas may shape their low gut bacterial diversity
Source: Conserv Physiol. 2020 Mar 13;8(1):coz104. doi: 10.1093/conphys/coz104 (PMC7066643; doi:10.1093/conphys/coz104)

Figure S1

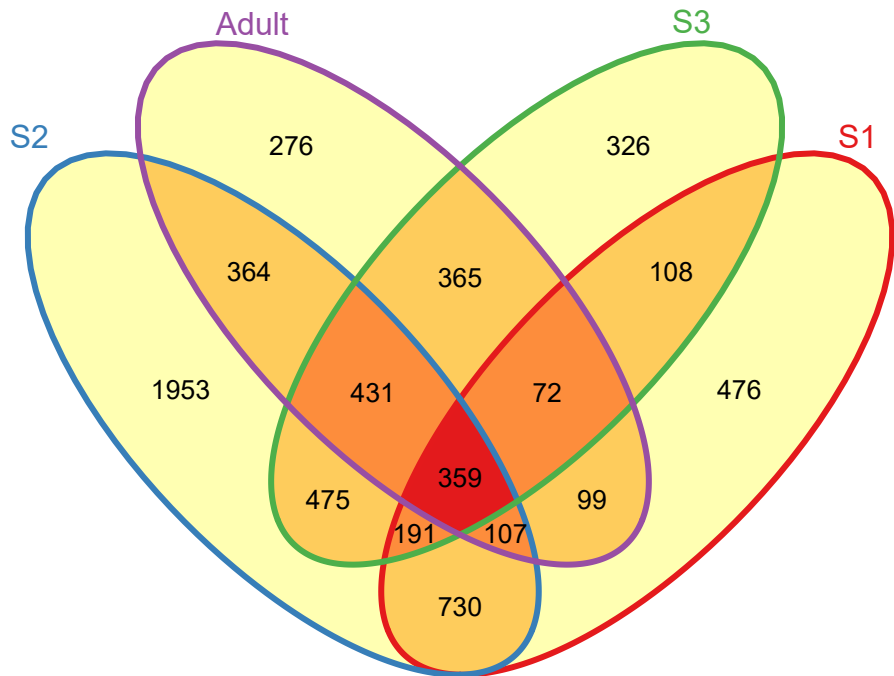

Supplement: figure_s1_coz104 [file figure_s1_coz104.pdf]

Figure S4

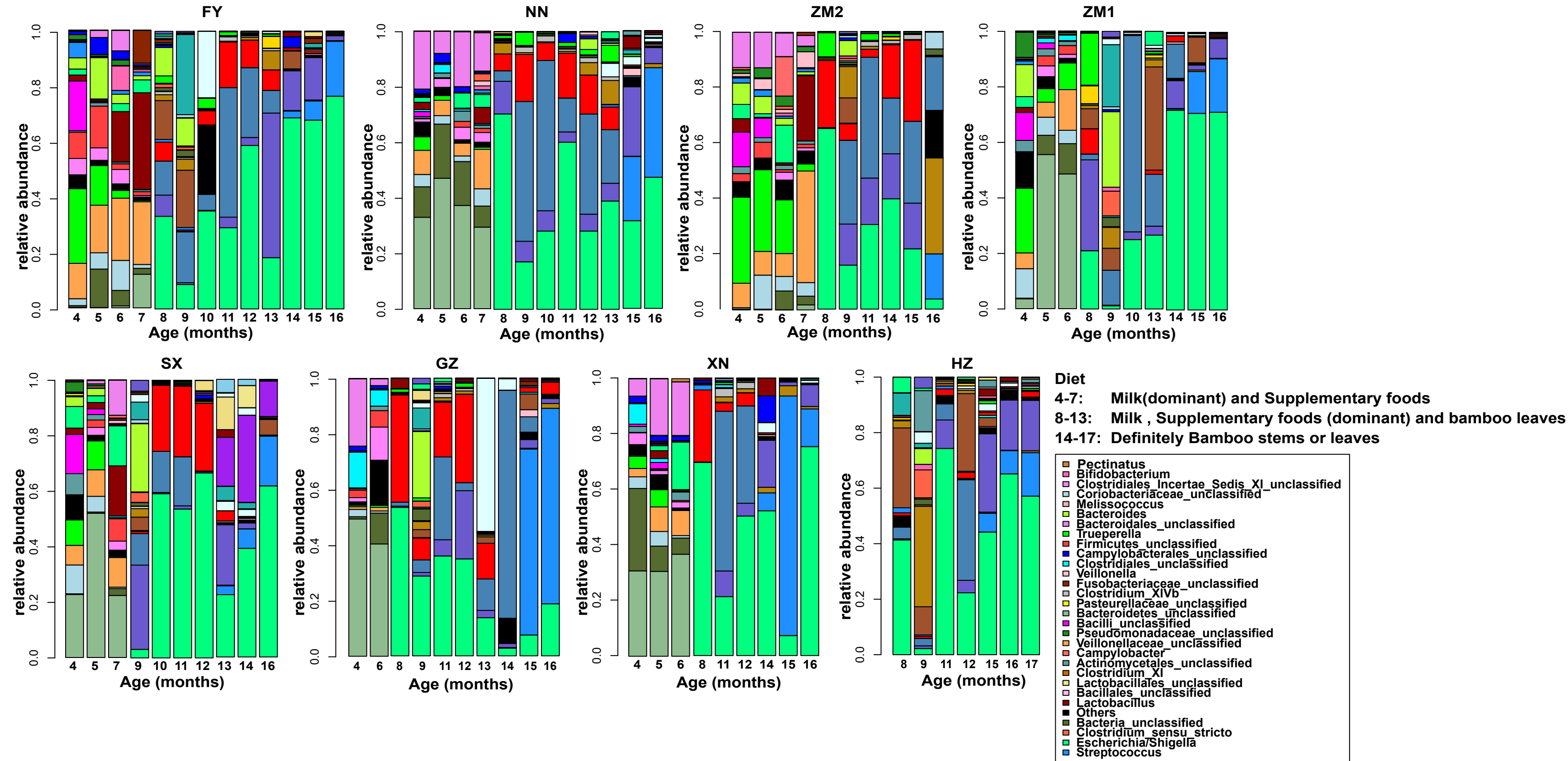

Supplement: figure_s4_coz104 [file figure_s4_coz104.pdf]

Figure S5

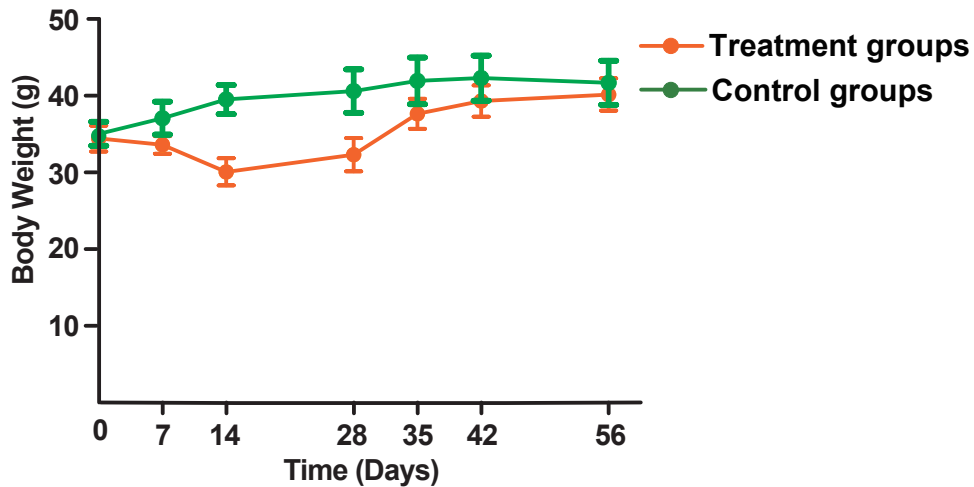

Supplement: figure_s5_coz104 [file figure_s5_coz104.pdf]
