# Supplementary material for: The carnivorous digestive system and bamboo diet of giant pandas may shape their low gut bacterial diversity
Source: Conserv Physiol. 2020 Mar 13;8(1):coz104. doi: 10.1093/conphys/coz104 (PMC7066643; doi:10.1093/conphys/coz104)
Supplement: figure_s2_coz104 [file figure_s2_coz104.pdf]

Figure S2

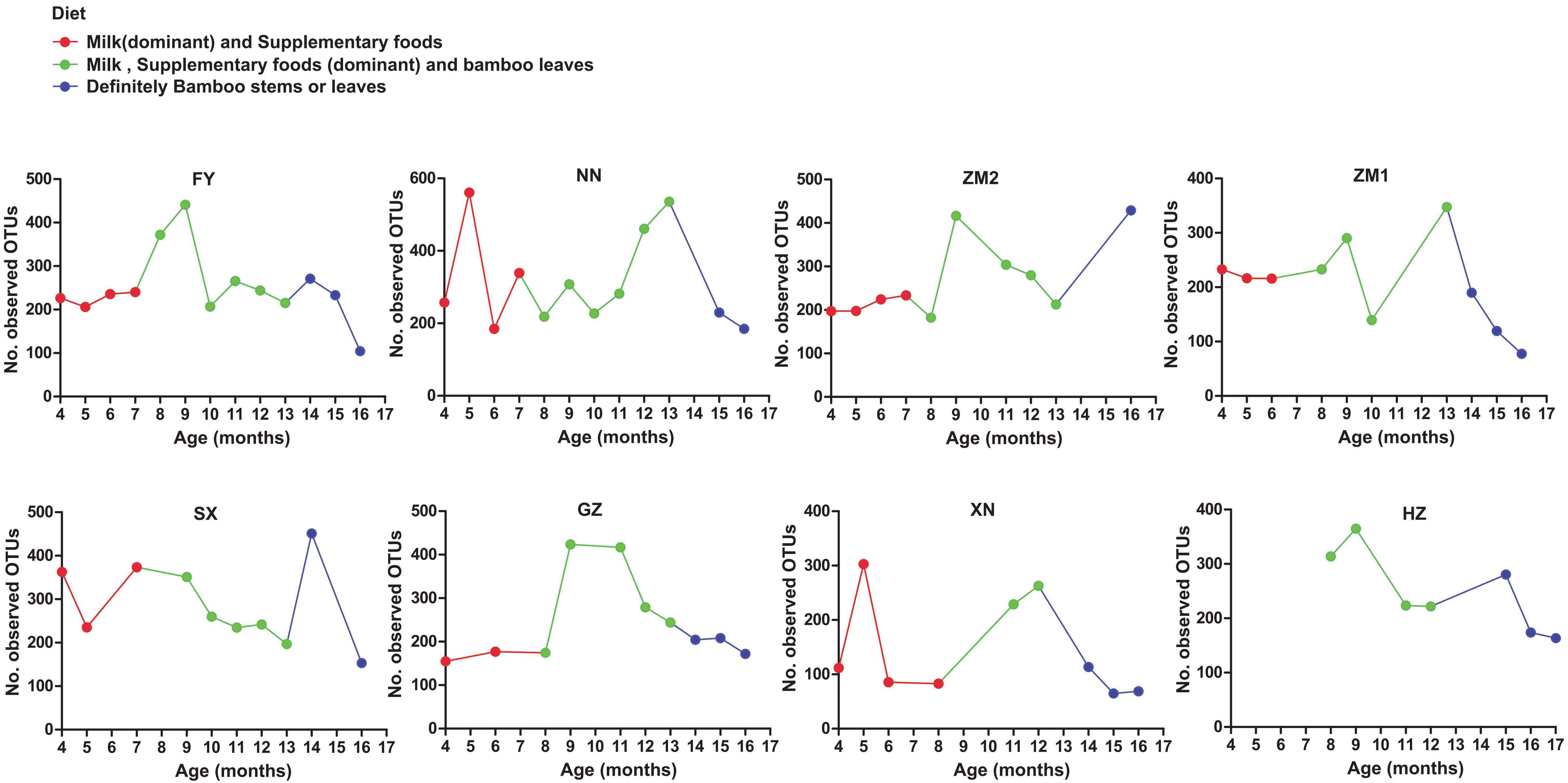

Diet
Milk(dominant) and Supplementary foods
Milk , Supplementary foods (dominant) and bamboo leaves
Definitely Bamboo stems or leaves
FY
NN
ZM2
ZM1
SX
GZ
XN
HZ
No. observed OTUs
Age (months)
