# Supplementary material for: The carnivorous digestive system and bamboo diet of giant pandas may shape their low gut bacterial diversity
Source: Conserv Physiol. 2020 Mar 13;8(1):coz104. doi: 10.1093/conphys/coz104 (PMC7066643; doi:10.1093/conphys/coz104)
Supplement: table_s2_coz104 [file table_s2_coz104.doc]

**Table S2. Nutritional informational of mice diet**

| Crude Protein | Crude Fat | Crude Fat | Crude Ash | Ca | P | Lys | Met+Cys |
| --- | --- | --- | --- | --- | --- | --- | --- |
| ≥ 20 % | ≥ 4 % | ≤ 5 % | ≤ 8% | 1.0-1.8 % | 0.6-1.2 % | ≥ 1.32 % | ≥ 0.78 % |

* Supplier: Chengdu Dashuo experimental animal co.LTD

** Product standard:GB14924-3-2010
