# Supplementary material for: The carnivorous digestive system and bamboo diet of giant pandas may shape their low gut bacterial diversity
Source: Conserv Physiol. 2020 Mar 13;8(1):coz104. doi: 10.1093/conphys/coz104 (PMC7066643; doi:10.1093/conphys/coz104)
Supplement: table_s3_coz104 [file table_s3_coz104.doc]

**Table S3. Sampling period and diet composition.**

| **Stage** | **Age** | **Diet** |
| --- | --- | --- |
| S1 | 4-7 months old | Milk(dominant) and Supplementary foods |
| S2 | 8-13 months old | Milk , Supplementary foods (dominant) and bamboo leaves |
| S3 | 14-17 months old | Definitely Bamboo stems or leaves |
| Adult | > 5 year old | Definitely Bamboo stems or leaves |

Supplementary foods including [steamed](javascript:void(0);) [corn](javascript:void(0);) [bread](javascript:void(0);), carrot and fruits etc
