# Supplementary material for: The carnivorous digestive system and bamboo diet of giant pandas may shape their low gut bacterial diversity
Source: Conserv Physiol. 2020 Mar 13;8(1):coz104. doi: 10.1093/conphys/coz104 (PMC7066643; doi:10.1093/conphys/coz104)
Supplement: supplemental_file_legends_coz104 [file supplemental_file_legends_coz104.doc]

**Additional files**

**Figure S1.** Venn diagram comparing OTUs in the different growth stages of giant pandas.

**Figure S2.** The dynamic curve of observed OTUs of gut microbiota in the different months of giant pandas.

**Figure S3.** The dynamic curve of Shannon indices of gut microbiota in the different months of giant pandas.

**Figure S4.** Relative abundance of OTUs at the genus level in the fecal microbiota of different growth stages of 8 giant panda cubs.

**Figure S5.** The body mass changes of mice during and after the experiment.

**Table S1.** Information and basic statistics on the additional datasets included in our comparative study.

**Table S2**. Nutritional informational of mice diet.

**Table S3.** Sampling period and diet composition.

**Table S4.** Detailed information of sampling and sequencing of 16S rDNA gene in this study.

**Table S5.** Results from group significance testing of individual OTUs.

**Table S6.** Detailed information of sampling and sequencing of 16S rDNA gene for mice in this study.
